# Evaluating key performance indicators of the process of care in juvenile idiopathic arthritis

**DOI:** 10.1186/s12969-023-00818-7

**Published:** 2023-04-21

**Authors:** Sarah M. Cooper, Gillian R. Currie, Seija Kromm, Marinka Twilt, Deborah A. Marshall

**Affiliations:** 1 Department of Community Health Sciences, Cumming School of Medicine, University of Calgary, Calgary, AB Canada; 2 Department of Paediatrics, Cumming School of Medicine, University of Calgary, Calgary, AB Canada; 3 Alberta Children’s Hospital Research Institute, University of Calgary, Calgary, AB Canada; 4 Health Research Innovation Centre, Room 3C56, 3280 Hospital Drive NW, Calgary, AB T2N 4Z6 Canada; 5 Maternal Newborn Child & Youth Strategic Clinical Network, Alberta Health Services, Calgary, AB Canada; 6 Section of Rheumatology, Department of Paediatrics, Cumming School of Medicine, University of Calgary, Calgary, AB Canada; 7 McCaig Institute for Bone and Joint Health, University of Calgary, Calgary, AB Canada; 8 O’Brien Institute for Public Health, University of Calgary, Calgary, AB Canada

**Keywords:** Key performance indicators, Quality improvement, Juvenile idiopathic arthritis

## Abstract

**Objective:**

To determine whether and how often the information to measure a set of key performance indicators (KPIs) in juvenile idiopathic arthritis (JIA) is found in data collected routinely in a Pediatric Rheumatology Clinic.

**Methods:**

A retrospective electronic chart review and administrative data analysis was conducted for a cohort of 140 patients with JIA at a tertiary Pediatric Rheumatology Clinic between 2016–2020. The set of KPIs include measuring patient outcomes (joint assessment, physician’s global assessment of disease activity, assessment of functional ability, composite disease activity measurement), access to care (waiting time between referral and first visit, visit with the rheumatologist within the first year of diagnosis, annual follow-up visits with the rheumatologist), and safety (tuberculosis screening, and laboratory monitoring). Documentation was assessed as a binary variable indicating whether the required information was ever found. Documentation frequency for each KPI was assessed with counts and percentages of the number of times the required information was documented for each clinic visit. Compliance with the safety KPI definitions was assessed using administrative databases.

**Results:**

Data for each KPI were found at least once in the cohort and documentation varied in frequency and consistency. Access to care and safety KPIs were documented more frequently than patient outcome KPIs. A joint assessment was documented at every visit for 95% of patients, 46% for an assessment of pain, and none for a physician’s global assessment of disease activity, an assessment of functional ability, or a composite disease activity measurement.

**Conclusion:**

Although feasible to measure, there is an opportunity for improving the consistency of documentation. Having an active system of monitoring KPIs and tools to simplify measurement is a key step in the process toward improved patient care outcomes. Streamlining the collection of KPI data can increase the likelihood of compliance. Next steps should involve replicating this study in various centres.

## Introduction

Juvenile idiopathic arthritis (JIA) is one of the most common chronic childhood rheumatic diseases [1]. Approximately 0.1% of children in Canada have JIA [2]. Without prompt diagnoses and treatment, patients with JIA experience persistent joint pain, swelling, and stiffness which leads to permanent disfigurement and disability. 

The Understanding Childhood Arthritis Network (UCAN) CURE team is a multicentre, international precision health program which examines biology-based treatment strategies for JIA. The UCAN CURE team developed a set of 10 key performance indicators (KPIs), consistent with current Canadian and international clinical guidelines, (and can be) used to assess the quality of care for JIA [3]. These indicators provide measurable parameters to assess processes, structures, and outcomes [4] that reflect the quality of care that a patient receives. KPIs provide data to support interventions which reduce “unwarranted variability” in practice and care[3, 5]. The UCAN CURE process KPIs are grouped into three categories: measurement of patient outcomes, access to care, and safety (Table 1). The access to care KPIs (visits during the first year of diagnosis, annual follow up visits, and waiting times for rheumatological consultation) were previously examined and gaps in waiting times for older JIA patients were identified [6, 7], but there have been no studies assessing the feasibility of measuring the full set of 10 JIA KPIs using routinely collected and readily available clinical data.

**Table 1:** UCAN CURE JIA KPI Definitions and Operational Definitions [3]

KPI	Definition	Operational Definition
**Measurement of Patient Outcomes KPIs**		
1. Rheumatological joint count	Percentage of patients where a joint count was conducted on the first visit and each subsequent visit using a validated tool	**Numerator**: the number of patients where a joint count was conducted on the first visit and each subsequent visit using any reliable tool in the measurement period **Denominator**: the total number of patients seen in the measurement period
2. Physician’s Global Assessment (PGA) of disease activity	Percentage of patients assessed for a PGA using any validated tool at the first visit and at each subsequent visit	**Numerator**: the number of patients assessed for a PGA at the first visit and at each subsequent visit in the measurement period **Denominator**: the total number of patients seen in the measurement period
3. Assessment of functional ability	Percentage of patients assessed for functional ability using any validated tool at the first visit and at every routine clinic visit	**Numerator**: the number of patients assessed for functional ability at the first visit and at each subsequent visit in the measurement period **Denominator**: the total number of patients seen in the measurement period
4. Composite disease activity measurement	Percentage of patients with an assessment of disease activity using the cJADAS^a^ at the first visit and at every routine clinic visit	**Numerator**: the number of patients with an assessment of disease activity using the cJADAS^a^ in the measurement period **Denominator**: the total number of patients seen in the measurement period
5. Assessment of arthritis-related pain	Percentage of patients assessed for pain at the first visit and each subsequent visit that occur at least 7-days apart using any validated age-appropriate tool to measure average pain	**Numerator**: the number of patients assessed for pain at the first visit and each subsequent visit using any validated, reliable, age-appropriate tool to measure average pain in the measurement period **Denominator**: the total number of patients seen in the measurement period
**Access to Care KPIs**		
6. Waiting times for rheumatologist consultation for patients with new onset JIA	The 50^th^ and 90^th^ percentile waiting time for rheumatologic consultation	**50**^**th**^** percentile**: the number of days that half the patients in the sample with new onset JIA saw a pediatric rheumatologist and half are still waiting **90**^**th**^** percentile**: the number of days that 90% of the patients in the sample with new onset JIA saw a pediatric rheumatologist and 10% are still waiting
7. Patients newly diagnosed with JIA with at least 1 visit to a pediatric rheumatologist in the first year of diagnosis	Percentage of patients with new onset JIA (incident JIA) with at least 1 visit to a pediatric rheumatologist in the first year of diagnosis	**Numerator**: the number of patients with new onset JIA with at least one visit to a pediatric rheumatologist in the first year of diagnosis during the measurement period **Denominator**: the total number of patients with new onset JIA seen during the measurement period
8. Patients seen in yearly follow-up by a pediatric rheumatologist	Percentage of patients with JIA seen by their pediatric rheumatologist at least once every year over	**Numerator**: the number of patients with a diagnosis of JIA under the case of a pediatric rheumatologist seen in follow up by a pediatric rheumatologist at least once every year during the measurement period **Denominator**: the total number patients with a diagnosis of JIA patients under the care of a pediatric rheumatologist in the measurement period excluding patients who meet exclusions
**Safety KPIs**		
9. Tuberculosis (TB) screening	Percentage of patients screened for TB within 12 months prior to receiving a first course of therapy using a biologic DMARD^b^	**Numerator**: the number of patients screened for TB within 12 months prior to start of any biologic therapy using a standard TB skin test/ blood test in the measurement period **Denominator**: the total number of patients on a biologic therapy in the measurement period
10. Laboratory monitoring for disease-modifying anti-rheumatic drugs (DMARDs)	Percentage of patients who received methotrexate and leflunomide and monitored for toxicity by clinical laboratory methods	**Numerator**: the number of patients who received methotrexate and leflunomide and monitored for toxicity 1 month after the start of therapy, and every 3–4 months after by clinical laboratory methods in the measurement period **Denominator**: the total number of patients who received methotrexate and leflunomide in the measurement period

^a^Clinical Juvenile Arthritis Disease Activity Score (cJADAS)

^b^Disease modifying anti-rheumatic drug (DMARD)

Evaluating the feasibility of measuring and reporting these KPIs will identify if these KPIs can be easily monitored using standard documentation in routine clinical visits. Having KPIs that are easily measured using readily collected data and then reported is a critical step toward transparency and accountability in delivering high quality patient care and improving patient outcomes. Measuring improvements for change in the quality of patient care can provide relevant information to induce quality improvements [8]. The objective of this study was to determine whether the required information to measure the set of JIA KPIs is found in data routinely collected in a Pediatric Rheumatology Clinic captured in the centralized electronic data capture system, and report on the frequency of documentation for each KPI.

## Patients and methods

### Cohort selection

Patients were identified from the Alberta Children’s Hospital (ACH) Pediatric Rheumatology Clinic, a tertiary practice and academic centre including seven pediatric rheumatologists providing care for pediatric patients up to (and including) the age of 17. The clinic provides multidisciplinary care; patients can access care from pharmacists, physiotherapists, registered nurses, rheumatologists, and social workers.

This project leveraged a dataset collected in a previous research study [9, 10], which identified a JIA cohort from the ACH using an administrative data algorithm [11] for JIA and confirmed diagnosis by cross-referencing JIA diagnosis in Calgary’s acute care electronic storage system – Sunrise Clinical Manager (SCM). When the diagnosis was unclear, the research team consulted a pediatric rheumatologist and patients were excluded if their diagnosis was secondary to another disease. The previous study chose the highest sensitivity case ascertainment algorithm to validate JIA diagnosis between 2011 and 2019. This two-step process was applied to increase the probability of all relevant patients being included in the cohort. As one visit would not adequately reflect a pattern of care for routinely collected data, each patient required a minimum of two clinic visits for inclusion into the cohort. Data collected within SCM consists of a summary of the clinic visit: consultation notes, nursing notes and pharmacy notes.

Of the 392 JIA patients at the ACH Pediatric Rheumatology Clinic, a subset of 140 patients met the inclusion criteria of having a JIA diagnosis and first visit with a pediatric rheumatologist at the clinic between January 1^st^, 2016, and December 31^st^, 2018. This period of diagnosis was chosen to provide the most recent KPI performance levels as well as sufficient time for follow-up to capture reporting patterns. Patients were followed from January 1^st^, 2016, to March 13^th^, 2020. Additional data were collected to complete the information required for each KPI listed in Table 1.

Ethics approval was obtained from The Conjoint Health Research Ethics Board, University of Calgary (REB19-0471).

### Data sources

#### Sunrise clinical manager

This study aims to measure the data captured on the centralised electronic system (Sunrise Clinical Manager (SCM)). Additional data may be captured outside of this system as part of routine research and clinical care, thus this is not a measurement of physician performance. Data for each KPI were extracted from AllScripts Sunrise Clinical Manager (SCM), the Calgary-wide electronic storage system used for medical records [12]. This system was chosen because it was the data source used in Calgary during the time of the study, and is the centrally accessible digitized summary of the in-clinic visits. SCM provides summary documents and clinical notes for each clinic visit, and the official record of the clinic visit. Information from the clinic visits is dictated by the physician (or other clinicians) and the visit letter is entered into SCM. There is also a central paper medical record at the hospital, which also receives the dictated letter, and may contain other non-clinic visit related information on the patient. In terms of in-clinic paper records, some other information may be stored that is not in SCM, for example of referral letters or some patient reported outcome measurements collected during routine clinical visits. SCM represents the official record of the clinical visit. This study focussed on the readily available electronic SCM data as the source document for clinic health visits, and did not consider other in-clinic paper records.

#### Administrative databases

The safety KPIs (#9 and #10) required information around tuberculosis (TB) screening and laboratory monitoring in addition to that found in SCM. This data was obtained from administrative databases: the Consolidated Laboratory Repository, Practitioner Claims, and the National Ambulatory Care Reporting System (NACRS). These three databases were used to determine whether patients had documentation in the datasets and the dates they were screened for TB (KPI #9). Practitioner Claims uses codes from the International Classification of Disease (ICD)-9; relevant codes were 795.7 and V74 and Health Service Code 98.8. NACRS uses ICD-10 codes; this study used code Z11.1 and Canadian Classification of Health Interventions (CCI) code 2ZZ08M. The Consolidated Laboratory Repository database was used to identify if patients on a biologic had a TB blood test and if patients on methotrexate and leflunomide had the relevant laboratory tests. Alberta’s Consolidated Laboratory Repository undergoes testing and data validation procedures before it is released for use by researchers [13].

There is an electronic source for referral letters, namely Clinibase, however, in this study, it was not used as it was only available after 2019 and all patients in this research were diagnosed between 2016 and 2018.

### Analysis

Data were first assessed and recorded as a binary (yes/no) variable based on the presence of data for each KPI being documented at least once in the cohort and then by the number of patients who had data for the respective KPI documented. The documentation frequencies were reported based on the operational definitions described in Table 1. For KPI definitions requiring a validated tool, data on the named tool was collected and then verified as a “validated tool” or not. The last visit at the clinic was defined as the earliest date when the patient either left the province, transitioned to adult care, or the March 13^th^, 2020, study cut-off date. For KPI #6, benchmarks for waiting times for a rheumatologist consultation of 7 days for systemic JIA and 4 weeks for the other JIA subtypes [7, 14] were used.

## Results

### Cohort description

As shown in Table 2, 57% of the JIA cohort were female and oligo-arthritis was the most frequent JIA subtype (41%). The median age at diagnosis was 11 years old. The median time between first visit with the pediatric rheumatologist and diagnosis date was 36 days. The shortest time between first pediatric rheumatologist visit and diagnosis date was 10 days prior to the first visit (two patients were diagnosed through hospital admissions before their first visit with the pediatric rheumatologist). The median time between symptom onset and first visit with the rheumatologist was four months and the average follow-up period (time between the first visit and the last visit at the clinic) was three years.

**Table 2:** Cohort demographics

	**Total Cohort (*n* = 140)**
**Patient Characteristics**	
Female, *n* (%)	80 (57%)
**JIA Subtype Groups**	
Systemic, *n* (%)	5 (4%)
Oligo-arthritis (persistent, extended, not specified), *n* (%)	57 (41%)
Polyarticular arthritis, *n* (%)	45 (32%)
Enthesitis-related arthritis, *n* (%)	24 (17%)
Other (psoriatic, undifferentiated, unknown), *n* (%)	9 (6%)
**Clinical Characteristics**	
Age at diagnosis (years)^a^	11 (6, 14), [1, 18]
Time between first PR^b^ visit and diagnosis date (days)^a^	36 (0, 103), [-10, 596]
Symptom onset^c^ (months)^a^	4 (2, 13), [0, 137]
Follow-up period^d^ (years)^a^	3 (2, 3), [0, 4]

No missing data points. All values rounded to the nearest whole number

^a^Median (Q1, Q3), [min, max]

^b^Pediatric rheumatologist (PR)

^c^Symptom onset: time between initial symptoms and first visit with pediatric rheumatologist

^d^Follow-up period: time between first visit at clinic and censored end of follow-up

### Documentation

All KPIs were documented at least once in the cohort. The safety KPIs were the most frequently captured electronically, followed by the access to care and then measurement of patient outcomes KPIs (Table 3). For the measurement of patient outcomes KPIs, 56 patients had a Childhood Health Assessment Questionnaire (CHAQ) score documented in at least one visit. No other assessment of functional ability was found in the SCM records. The explicit clinical juvenile arthritis disease activity score (cJADAS) was also not found in SCM; however, 58 patients had the components required to calculate the cJADAS (joint count, physician’s global assessment (PGA), and parent/patient assessment of well-being) documented in at least one visit. The most documented of the five measurement of patient outcomes KPIs were rheumatological joint assessment and assessment of arthritis-related pain. There was no specific validated tools documented for the joint or pain assessments. The PGA was documented in at least one visit for 47% of the patients. For the three access to care KPIs, visit dates for first year diagnosis and follow-up visits were the most frequently documented. Of the two safety KPIs, laboratory monitoring for patients on the DMARDs methotrexate and leflunomide was documented in all patients; however, TB screening was documented in 96% of the patients. Although this information is a low first hurdle for KPI reporting, it gives the opportunity to assess the documentation frequencies found in the next section.

**Table 3:** Documentation of UCAN CURE KPIs in SCM and the administrative databases

KPI	Documented^a^ in at least one visit for each patient, n (%)
**Measurement of Patient Outcomes KPIs**	
1. Rheumatological Joint Assessment in SCM	140 (100%)
2. Physician's Global Assessment of Disease Activity in SCM	66 (47%)
3. Assessment of Functional Ability	56 (40%)
Assessment of Functional Ability using CHAQ in SCM	56 (40%)
Other Assessment of Functional Ability in SCM^b^	0
4. Composite disease activity measurement	58 (41%)
All Components of cJADAS present in SCM	58 (41%)
cJADAS in SCM^c^	0
5. Assessment of arthritis-related pain in SCM	140 (100%)
**Access to Care KPIs**	
6. Waiting time between referral date and first visit in SCM	24 (17%)
7. Presence of visit dates for first year of diagnosis in SCM [*n* = 137]^d^	137 (100%)
8. Presence of visit dates for follow-up visits in SCM [*n* = 137]^d^	137 (100%)
**Safety KPIs**	
9. Tuberculosis Screening [*n* = 56]^e^	54 (96%)
Tuberculosis Screening in SCM [*n* = 56]^e^	53 (95%)
Tuberculosis Screening in Consolidated Laboratory Repository [*n* = 56]^e^	5 (9%)
Tuberculosis Screening in Practitioner Claims [*n* = 56]^e^	18 (32%)
Tuberculosis Screening in NACRS [*n* = 56]^e^	4 (7%)
10. Laboratory Monitoring for DMARDs^f^ [*n* = 102]^g^	102 (100%)
Labs Ordered in SCM [*n* = 99]^h^	91 (92%)
Lab Results in SCM [*n* = 99]^h^	91 (92%)
Laboratory Tests in Consolidated Laboratory Repository [*n* = 102]^g^	102 (100%)

^a^ ‘Documented’ – means data required for each KPI is found in at least one visit in the entire JIA cohort. If the data were found to be documented, this is shown in the table as ‘Yes’. If data were not found, this is shown in the table as ‘No’. If the KPI is not relevant to be reported separately for the respective visit type, it is shown in the table as N/A

^b^The CHAQ was the only assessment of functional ability found

^c^The cJADA score was never explicitly found, only the components required to calculate the cJADAS (joint count, physician’s global assessment, parent/patient assessment of well-being)

^d^*n* = 137 because 3 patients did not have a follow-up visit after diagnosis date

^e^*n* = 56 because this is only applicable to those prescribed biologics. Documentation of tuberculosis screening in any of the data sources used. One patient’s screening was not documented in SCM but was documented in the Consolidated Laboratory Repository

^f^Disease modifying anti-rheumatic drugs (DMARDs)

^g^*n* = 102 because this is only applicable to those prescribed the DMARDs methotrexate and leflunomide

^h^*n* = 99 because this is only applicable to patients who had a visit after being prescribed the DMARDs methotrexate and leflunomide

### Documentation frequencies

#### Measurement of patient outcomes KPIs

Rheumatological joint assessment was the most documented KPI, occurring at almost every visit for every patient (95%) and was only missed in one patient chart at the first visit (Table 4). This documentation frequency is considerably higher than the PGA of disease activity (only 15% of visits). The CHAQ was the only method of assessment for functional ability found in SCM and was documented in at least one visit for less than half the cohort. The assessment of arthritis-related pain had one of the highest documentation frequencies for the number of total and first visits with documentation (Table 4). Documentation of this KPI dropped over time as the percentage of patients with documentation at every visit was less than half that for the first visit. The data were not able to determine if a validated tool to assess pain and a joint assessment were used as no named tools were specified.

**Table 4:** Documentation frequency of measurement of patient outcomes KPIs found in SCM

KPI	Number of visits with documentation, n (%) [*n* = 1360]	Number of first visit with documentation, n (%) [*n* = 140]	Number of patients with documentation at every visit [*n* = 140]
**Measurement of Patient Outcomes KPIs**			
Rheumatological Joint Assessment in SCM	1351 (99%)	139 (99%)	133 (95%)
Physician’s Global Assessment of Disease Activity in SCM	205 (15%)	1 (1%)	0
Assessment of Functional Ability	150 (11%)	1 (1%)	0
Assessment of Functional Ability using CHAQ in SCM	150 (11%)	1 (1%)	0
Other Functional Ability Assessment in SCM^a^	0	0	0
Composite disease activity measurement	169 (12%)	1 (1%)	0
All Components of cJADAS present in SCM	169 (12%)	1 (1%)	0
cJADAS in SCM^b^	0	0	0
Assessment of arthritis-related pain in SCM	1186 (87%)	124 (89%)	65 (46%)

^a^The CHAQ was the only assessment of functional ability found in SCM

^b^The cJADAS was never explicitly found, only the components required to calculate the score (joint count, physician’s global assessment, parent/patient assessment of well-being)

#### Access to care KPIs

For 24 patients, the time from referral date to first visit at the clinic was mentioned in the documentation found in SCM either numerically or with a qualitative description where a date was not able to be determined or calculated (Table 5). Of these patients, 18 had a quantitative timeframe mentioned and only one patient had an explicit referral date reported. For the remaining 17 patients, when the referral date was not explicit, references within the documentation were used to determine the date of referral; when only the month and not the day was reported, the 15^th^ day of the month was used for the referral date. The cohort size for systemic JIA patients was too small to report independently. There were 14 systemic and non-systemic JIA patients that met the respective benchmarks of seven-days for systemic JIA and four-weeks for the other JIA non-systemic subtypes. For patients with non-systemic JIA, waiting time for rheumatologic consultation, defined as the 50^th^ and 90^th^ percentile, was 24 days and 45 days respectively.

**Table 5:** Documentation frequency of access to care KPIs

KPI	N (%)
**Access to Care KPIs**	
Waiting times for rheumatologist consultation for patients with new onset JIA	
Number of patients with a qualitative or quantitative documentation for waiting times [*n* = 140]	24 (17%)
Number of patients with an eligible date of referral reported [*n* = 24]^a^	18 (75%)
Number of patients that met benchmark of time from referral to first visit in days, [*n* = 18]^b^	14 (78%)
50th percentile in days, [*n* = 18]	24
Non-systemic JIA patients in days, [*n* = 16]	24
90th percentile in days, [*n* = 18]	46
Non-systemic JIA patients in days, [*n* = 16]	45
Patients newly diagnosed with JIA with at least 1 visit to a pediatric rheumatologist in the first year of diagnosis	
Number of patients with new onset JIA (incident JIA) with at least one visit to a pediatric rheumatologist in the first year of diagnosis [*n* = 137]	137 (100%)
Patients seen in yearly follow-up by a pediatric rheumatologist	
Number of patients with JIA seen by their pediatric rheumatologist at least once every year over their follow-up period [*n* = 137]	105 (77%)
12 to 24 months after diagnosis [*n* = 128]	123 (96%)
24 to 36 months after diagnosis [*n* = 89]	74 (83%)
36 to 48 months after diagnosis [*n* = 36]	24 (67%)

^a^Includes approximate date used for eligible date of referral reported and complete date of referral reported. When no specific date was reported and only month/year, 15^th^ of the month was used

^b^Benchmark is 7 days for systemic JIA and 4 weeks for other types of JIA and denominator is *n* = 140

The KPI for visits during the first year of diagnosis, defined as 12 months after diagnosis, had a sample size of 137 because three patients did not have a visit date after their date of diagnosis (two patients transitioned to adult care and one patient was lost to follow-up after diagnosis). Of these patients who had at least one visit after diagnosis, 100% saw the pediatric rheumatologist in the first 12 months after diagnosis.

Patient-centric years, defined by the anniversary of the JIA diagnosis date, were used to calculate annual follow-up visits with the pediatric rheumatologist after the first 12-month period. Over each patient’s follow-up period, 77% (105 of the 137) had a visit during each eligible interval. To be included in the denominator, patients must have visited a pediatric rheumatologist after their date of diagnosis. In the 12- to 24-month and 24- to 36-month intervals after diagnosis, 96% and 83% of patients had at least one follow-up visit. In the 36- to 48-month interval after diagnosis, 67% of patients had at least one visit with a rheumatologist. The lower performance of the 36- to 48-month interval after diagnosis is likely due to the 12-month window for some patients [*n* = 36] extending past the study’s ultimate cut-off date of March 13^th^, 2020.

#### Safety KPIs

In the cohort, 56 patients were on a biologic with 71 biologic starts documented in SCM. This is because some of the 56 patients on a biologic had taken multiple biologics over the course of their follow-up period, thus increasing the number of ‘starts’. Documentation for TB testing in either SCM, the consolidated laboratory repository, NACRS, or Practitioner Claims was found for 96% of the patients. Of the 56 patients, 53 patients had a documented TB test for a biologic start in SCM (Table 6). Screening for TB can take many different forms such as a blood test, skin test, or chest x-ray. We found that documentation in SCM did not include the date the TB test was completed; therefore, an analysis was reported separately for documentation in each applicable data source and then the number of patients who had a TB test documented within 12 months prior to receiving their first course of biologic therapy. Since a time-component was required for the KPI definition and a time-component for when the TB test occurred was not able to be determined using SCM, this analysis was reported separately. In total, five patients met the TB screening KPI (screening within 12 months prior to first biologic), two patients were found with documentation in NACRS, and three patients were found with documentation in Practitioner Claims.

**Table 6:** Documentation frequency of safety KPIs in SCM and the administrative databases

KPI	N (%)
**Safety KPIs**	
Tuberculosis Screening	
Number of patients with documentation of a TB test for a biologic start in SCM, Practitioner Claims, NACRS, or Consolidated Laboratory Repository [*n* = 56], n (%)	54 (96%)
SCM [*n* = 56]	53 (95%)
Practitioner Claims [*n* = 56]	18 (32%)
NACRS [*n* = 56]	4 (7%)
Consolidated Laboratory Repository [*n* = 56]	5 (9%)
Number of patients screened for TB within 12 months prior to receiving a first course of therapy using a biologic DMARD [*n* = 56]	5 (9%)
Practitioner Claims [*n* = 18]	3 (17%)
NACRS [*n* = 4]	2 (50%)
Consolidated Laboratory Repository [*n* = 5]	0
Laboratory monitoring for DMARDs	
Number of patients on methotrexate and leflunomide with documentation of toxicity monitoring in the Consolidated Laboratory Repository [*n* = 102]	102 (100%)
0–1 month [*n* = 102]	60 (59%)
1–4 months [*n* = 101]	85 (84%)
4–8 months [*n* = 94]	85 (90%)
8–12 months [*n* = 88]	77 (88%)
12–16 months [*n* = 79]	70 (89%)
16–20 months [*n* = 68]	53 (78%)
20–24 months [*n* = 55]	42 (76%)
Number of patients who received methotrexate and leflunomide and monitored for toxicity by clinical laboratory methods in the Consolidated Laboratory Repository during every eligible interval [*n* = 102]	29 (28%)
Number of patients on methotrexate or leflunomide with mention of laboratory tests ordered^b^ in SCM at every eligible visit [*n* = 99]^a^	22 (22%)
Number of patients on methotrexate or leflunomide with mention of laboratory test results^c^ in SCM at every eligible visit [*n* = 99]^a^	33 (33%)

^a^Does not include patients who began methotrexate or leflunomide at last visit and denominator is entire cohort. ^b^Mention of laboratory tests ordered includes documentation reporting specific tests, non-specific tests, and ordering no tests

^c^Mention of laboratory test results includes documentation reporting specific tests, missing/pending/not yet available tests, non-specific tests, and documentation that no recent tests were completed

In the cohort, 102 patients were on the DMARDs methotrexate or leflunomide with a documented laboratory monitoring test (Table 6). Documentation in SCM required the patient to have a visit during the follow-up period after being prescribed a DMARD, thus the sample size for documentation in SCM was 99 patients. During the first month the patient received methotrexate or leflunomide, 59% of eligible patients were monitored for toxicity by clinical laboratory methods in the Consolidated Laboratory Repository. Focusing on the first two-years of eligible intervals, the percentages of patients who received methotrexate and leflunomide and were monitored for toxicity are high, ranging from 76 to 90% after the first month. The high level of testing is in accordance with the mean duration a patient was on the drug (22 months).

## Discussion

The access to care and safety KPIs were documented more frequently than the measurement of patient outcomes KPIs. In terms of overall documentation for the patient outcomes KPIs, the joint and pain assessment KPIs (#1 and #5) were documented more than 80% of the time which is in line with benchmarks proposed by Lovell et al.[15] and can be easily used in future analyses. Although the current documentation frequencies for each of the 10 JIA KPIs are sufficient to develop benchmarks of care, there is a significant opportunity for better clinical documentation and more consistent data collection for KPIs during clinical visits, which aligns with current clinical guidelines for JIA management [3].

A joint assessment was the most frequently documented KPI in this study. The standardized layout of the SCM form which includes physical examination having its own section with a description of a joint assessment likely facilitated this finding. It is unknown whether the frequent documentation of this KPI will continue with the transition to Epic (a new comprehensive electronic health record (EHR) being implemented across Alberta)[16, 17].

The data for patient outcomes KPIs (physician’s global assessment (PGA), assessment of functional ability, and measurement of clinical disease activity) were minimally documented in SCM. As noted earlier, Childhood Health Assessment Questionnaire (CHAQ) and other patient reported outcome measurements are currently documented in clinic paper charts. Moving to Epic’s electronic system may provide an opportunity to increase the frequency of documented CHAQ and PGA values if the assessments can be completed electronically rather than requiring a transfer of information from paper forms to the EHR.

In a prospective UK JIA study, data for cJADAS 10 (active joint count > 10 is given 10 points) were available for 96%, 77%, 94%, 87% and 80% at baseline, 6-month, year 1, year 2, and year 3 follow-ups respectively [18]. However, the UK study excluded patients if no cJADAS score could be calculated at any point. This could explain the large difference in data availability between the UK study and the present study.

In a previous study on rheumatoid arthritis (RA), a disease activity performance measure was defined as the “percent of RA patients with ≥ 50% of total number of outpatient encounters per year with assessment of disease activity using a standardized measure”, and 100% of the patients met this measure [19]. This contrasts dramatically to this study with only 12% of all JIA clinic visits documenting the cJADAS in SCM. It is possible that the higher levels of reporting disease activity by any acceptable composite measure (such as Disease Activity Score 28 or the Clinical Disease Activity Index) in the previous RA study was due to the use of the data platform Rheum4U, developed for inflammatory disease patients and implemented in both clinics in the study, with a patient platform to collect the patient reported outcomes. These higher levels could mean that the data are not routinely documented unless part of a specific RA registry where patient outcome data are explicitly recorded. The ease of monitoring when the required data for each KPI are entered into a platform that retrieves data for arthritis patients from selected electronic health records (EHR) systems such as Epic should be a key priority for the implementation of performance measures.

Epic is one of the EHRs commonly used with the Rheumatology Informatics System for Effectiveness (RISE) registry in the United States [20]. The registry automatically collects data from EMR and helps clinicians monitor quality of care by tracking performance at the patient-level on various measures as well as allow clinicians to compare themselves to their peers nationally [21]. A study using the RISE registry for RA found a performance rate for disease activity of 55.2% [22] and another found a performance of 53.6% in a random sample of RA patients [23] with the same KPI definition of documentation in ≥ 50% of outpatient encounters per year. The documentation of these KPIs in our context,could be improved with a streamlined transition of data from the paper chart to the electronic note. This could be facilitated through standardized headers for each clinician note, requiring data to be entered before the form can be completed, and having the software automatically calculate scores for various assessment such as the cJADAS.

The assessment of arthritis-related pain had a high documentation frequency for the total number of visits in the cohort but dropped in frequency for every visit for every patient. This could be because there was no standard section for pain similar to the physical examination section for joint assessment. Pain assessment is typically written at the start of the note where anything that has occurred since the last clinic visit is described and it is possible that the pain information does not get transferred to SCM if the patient’s pain was not significant in that visit. A specific section for pain in the SCM notes would be a step toward improved documentation patterns. Adoption of standardized measurements for pain in JIA could also help in this regard. In addition to the pain visual analog scale in the CHAQ, there are other available validated tools for pediatric use in JIA, such as the SUPER-KIDZ tool and the Iconic Paint Assessment Tool (IPAT) [24].

Documentation of the access to care KPIs was highly compliant except for the waiting times KPI. In SCM, the waiting time from referral was only mentioned in 17% of the cohort. After 2019, another database (Clinibase) contained the referral letters for each patient however as noted earlier our cohort was diagnosed between 2016–2018. Visits during the first year of diagnosis and annual visits demonstrate strong compliance as all applicable patients had a visit during the first year after diagnosis and 77% of patients had annual follow-up visits. It is possible that performance is higher if some visits were not entered into SCM.

Of the two safety KPIs, tuberculosis (TB) screening was documented in SCM more consistently than the KPI for laboratory monitoring for DMARDs. The TB screening KPI was documented in SCM for 95% of eligible patients. A noted limitation with the SCM data is that the dates of TB tests were not recorded; consequently, it could not be determined if TB testing occurred prior to the patient’s biologic therapy unless documented in the administrative databases. The Consolidated Laboratory Repository contains the tests for patients who received a TB screening blood test. This is typically only done for patients who recently had vaccinations or had prior TB exposure. This would explain the small number of patients who have this test reported in the Consolidated Laboratory Repository. The TB skin test is an ACR recommended screening test for latent TB and is the predominately used test for TB screening in Calgary [25]. NACRS or Practitioner Claims were used to identify TB screening; however, it has been consistently found that using ICD codes to identify TB screening and diagnoses has a relatively lower positive predictive value compared to other communicable diseases such as meningococcal meningitis and pneumococcal meningitis [26]. A TB skin test can also be performed in the Infectious Disease Clinic at the hospital may not appear in the NACRS data. A more accurate method of identifying TB screening should be a focus moving forward.

Although the laboratory monitoring KPI for patients on methotrexate and leflunomide was well documented in SCM, the Consolidated Laboratory Repository contained more accurate data. Even so, this study was unable to determine the exact biologic start date without patient interaction documented. Accuracy for start and stop dates could be improved by using the Pharmaceutical Information Network database to track the date the prescription was dispensed, patient feedback on their start and stop dates, or having more explicit description headers in the clinic visit note. The lower compliance levels for laboratory testing demonstrates that there is an opportunity for improvement in compliance and documentation of this KPI.

Quality measurement is dependent upon the availability of relevant data. This was cited as the greatest factor that facilitated or impeded the use of quality measures by the National Quality Forum report [27]. Data infrastructures need to be able to “talk to each other” and EHRs need to be “sufficiently robust” to generate the required information for “measure construction” [27]. It has been shown that performances in practices are the highest when the EHR system has rheumatology-specific templates in the software as it enables the collection and monitoring of key measures [28]. EHRs should be used to guide which process-related quality indicators are easily assessed in clinical care [29]. An important next step in the implementation of these KPIs is to align the measures across Canada and have them endorsed by the Canadian Rheumatology Association. Implementing nationally aligned and endorsed measures with a system similar to RISE or Rheum4U in Epic would provide the highest likelihood of physician uptake and potential for quality improvement.

There are four data sources for performance measurement in health care: administrative data, chart review (paper and electronic documents), surveys of patients/families/staff, and data generated and extracted from EHRs [30]. Measurement using administrative data necessitates the assumption that the diagnosis and procedure coding is accurate and medication that was prescribed matches the medication taken [30]. Chart reviews are labour intensive and used to validate measures from administrative data and EHRs [30]. The use of electronic health records provides an “opportunity to access patient-centric clinical data and the ability to efficiently measure quality performance outcomes measures” [30]. Technological advances have enabled data extraction from both discrete and free-text fields in EHRs [30]. Calgary’s new system, Epic, has the potential to capture the required data from a variety of data locations and consolidate to a single electronic database system, increasing the ease of monitoring KPIs by physicians and decision makers.

In addition to the previously mentioned RISE registry in the U.S., there are other international efforts being made to link clinical registries to electronic health records, and generate core minimal datasets. The CAPTURE-JIA (Consensus derived, Accessible (information), Patient-focused, Team-focused, Universally-collected (UK), Relevant to all and containing Essential data items) electronic dataset is in the pilot phase for determining the feasibility of data collection and moving towards a core national dataset in the UK [31, 32]. In the EU there are also endeavors in generating core minimal datasets that look at core elements of datasets to “facilitate better co-operative use of such data sets for research and health system administration.”[33] For example, a core dataset in juvenile dermatomyositis was developed for clinical settings that can later be incorporated into larger registries at the national and international level [34]. Local efforts of standardized data collection for minimal core datasets should align with international efforts to allow for global research collaboration to improve disease understanding. The glossaries accompanying these minimal core datasets can also be helpful in clinician training for those less familiar of the standardisation of data collection for the accompanying disease [34].

## Strengths and Limitations

This study used a retrospective cohort which minimized the risk of the Hawthorne effect as no clinicians were able to change their behaviour in response to the study. A standardized data collection form was used, and double data extraction occurred in 10% of the patients to minimize any discrepancies. The Alberta Children’s Hospital Pediatric Rheumatology Clinic is a tertiary centre, where clinicians are involved in research and are likely aware of quality measures and the value of documenting their JIA management with respect to clinical guidelines. Although this represents a strength in the data being more completely documented, it limits the generalizability of the results to other centres. There is a possibility that the data required for the KPIs is being collected but is not being documented in SCM. There may also be a potential concern that data are being documented in the clinical notes but not in a standardized format leading to data not being captured in the data collection form for this research. Referrals to multidisciplinary teams and outpatient services were not documented or captured in this study. It is important that ensuring transparency and accountability in delivering high quality patient care follows all access to medical services beyond the pediatric rheumatologist. This is something that should be implemented in future research on KPIs in JIA.

Further research is required to assess the feasibility and performance of these KPIs in other practice settings since there may be differences in how documentation of patient data are captured and stored. This objective of this research was to assess the data that were captured electronically in SCM and the readily available administrative databases. The interpretations from this research are based on the extent to which the required data are documented accurately in SCM. The study does not address, infer, or imply any evaluation of physician performance.

As health systems transition to electronic health records like Epic, efforts should be made to guide consistent data collection. It is important to understand the documentation patterns in relevant and routinely collected data to assess the feasibility of measuring predetermined KPIs. If KPIs are not measurable, they will be challenging to implement, limiting the potential for quality improvement and practice change. Individuals and systems need the tools to enact change and having an active system of monitoring KPIs that are feasible to measure is a key step in the process toward improved patient care outcomes. Effective monitoring of quality of care by streamlining and integrating the collection of data required to measure KPIs can help increase the likelihood of clinician uptake.

Future steps should involve prospective studies at centres where new electronic automated systems have been implemented and reviewing the documentation frequency results with similar experts to those involved in the development of the KPIs (Modified Delphi Panel) to determine if another step to update and confirm the KPIs is necessary before finalizing them [35]. Then, dissemination of the KPIs to clinicians involved in JIA patient care should occur to bring awareness to what data needs explicit documentation to allow for measurability and monitoring through engagement with Canadian rheumatologists. The final step to monitor these KPIs is to generate an algorithm to monitor, measure, and publicly publish the KPI levels at various JIA care centres to make them easily accessible for decision-makers.

## Conclusion

The results from this research could help drive quality improvement efforts through the dissemination to clinicians involved in JIA patient care to bring awareness to what data needs explicit documentation to allow for measurability and monitoring. By understanding what data are currently and consistently being captured in SCM that contains free-text fields rather than data-discrete fields, this research provides a focus to clinicians in rheumatology clinics about what data needs more to be captured electronically more consistently This research also applies to other sites that use AllScripts or other electronic medical records.

## Data Availability

The data that support the findings of this study are available from Alberta Health Services, but restrictions apply to the availability of these data, which were used under license for the current study, and so are not publicly available. Data are however available from the authors upon reasonable request and with permission of Alberta Health Services.

## Abbreviations

JIA: Juvenile idiopathic arthritis
KPI: Key performance indicator
ACH: Alberta Children’s Hospital
SCM: Sunrise Clinical Manager
TB: Tuberculosis
NACRS: National Ambulatory Care Reporting System
ICD: International Classification of Disease
CCI: Canadian Classification of Health Interventions
CHAQ: Childhood Health Assessment Questionnaire
cJADAS: Clinical juvenile arthritis disease activity score
PGA: Physician’s global assessment
DMARD: Disease modifying anti-rheumatic drug
EHR: Electronic health record
RISE: Rheumatology Informatics Systems for Effectiveness
EAC: Early arthritis clinic
RA: Rheumatoid arthritis
ACR: American College of Rheumatology
